# The bidirectional tumor - mesenchymal stromal cell interaction promotes the progression of head and neck cancer

**DOI:** 10.1186/scrt484

**Published:** 2014-08-12

**Authors:** Benjamin A Kansy, Philip A Dißmann, Hatim Hemeda, Kirsten Bruderek, Anna M Westerkamp, Vivien Jagalski, Patrick Schuler, Katinka Kansy, Stephan Lang, Claudia A Dumitru, Sven Brandau

**Affiliations:** Department of Otorhinolaryngology, University Hospital Essen, Essen, Germany; Helmholtz-Institute for Biomedical Engineering, RWTH Aachen University Medical School, Aachen, Germany; Department of Oral and Maxillofacial Surgery, University Hospital Heidelberg, Heidelberg, Germany

## Abstract

**Introduction:**

Mesenchymal stromal cells (MSC) are an integral cellular component of the tumor microenvironment. Nevertheless, very little is known about MSC originating from human malignant tissue and modulation of these cells by tumor-derived factors. The aim of this study was to isolate and characterize MSC from head and neck squamous cell carcinoma (HNSCC) and to investigate their interaction with tumor cells.

**Methods:**

MSC were isolated from tumor tissues of HNSCC patients during routine oncological surgery. Immunophenotyping, immunofluorescence and *in vitro* differentiation were performed to determine whether the isolated cells met the consensus criteria for MSC. The cytokine profile of tumor-derived MSC was determined by enzyme-linked immunosorbent assay (ELISA). Activation of MSC by tumor-conditioned media was assessed by measuring cytokine release and expression of CD54. The impact of MSC on tumor growth *in vivo* was analyzed in a HNSCC xenograft model.

**Results:**

Cells isolated from HNSCC tissue met the consensus criteria for MSC. Tumor-derived MSC constitutively produced high amounts of interleukin (IL)-6, IL-8 and stromal cell-derived factor (SDF)-1α. HNSCC-derived factors activated MSC and enhanced secretion of IL-8 and expression of CD54. Furthermore, MSC provided stromal support for human HNSCC cell lines *in vivo* and enhanced their growth in a murine xenograft model.

**Conclusions:**

This is the first study to isolate and characterize MSC from malignant tissues of patients with HNSCC. We observed cross-talk of stromal cells and tumor cells resulting in enhanced growth of HNSCC *in vivo*.

## Introduction

Mesenchymal stromal cells (MSC) are multipotent, fibroblast-like progenitor cells that can differentiate into multiple lineages, including osteogenic, adipogenic and chondrogenic cell types. MSC express specific cell surface markers such as CD29, CD73, CD90 and CD105 while lacking hematopoietic markers such as CD14, CD34 and CD45 [1]. Initially described as multipotent stromal cells originating from the bone marrow, MSC were later isolated from various fetal and adult tissues, including the salivary gland, placenta, umbilical cord blood, muscle, adipose tissue, connective tissue and peripheral blood [2–7]. Recently, MSC were also identified in pathological tissues and were found to be actively recruited towards tumors and inflammatory microenvironments [8–11].

The functions of MSC within the tumor microenvironment are very complex and still require extensive characterization [12]. For instance, MSC can differentiate into endothelial or vascular smooth muscle cells and might be critically involved in angiogenic processes [13]. Furthermore, MSC might have strong immunoregulatory effects on both innate and adaptive immune cells as they were shown to modulate the proliferation of T cells, B cells or natural killer cells by releasing various cytokines and metabolites of the arachidonic acid pathway [14–16]. The strong modulatory potential of MSC is further highlighted by their ability to release either proinflammatory or anti-inflammatory factors in response to different types of stimulation [17]. Altogether, these findings indicate that MSC engage in a multidirectional communication with the other cells of the microenvironment and are likely to play an important role in the progression of solid tumors.

Indeed, a number of recent studies demonstrated that MSC enhanced tumor growth and metastasis in several types of cancer [9, 18–23]. MSC exerted these tumor-promoting effects by enhancing angiogenesis, inducing immunosuppression or inhibiting the apoptosis of tumor cells [22, 24, 25]. In sharp contrast, other studies found that MSC had potent antitumor effects by inhibiting angiogenesis, promoting antitumor immune responses and inducing apoptosis of the cancer cells [26–33]. A possible explanation for this discrepancy may be the variability of MSC depending on the tissue and the microenvironment from which they were isolated [34]. Additionally, some variability might exist between human MSC and MSC derived from murine tissues – which have been used in many of the previous studies. Because of these caveats, the exact effects of human tumor-resident MSC on the progression of human tumors remain unclear and need to be further investigated.

In this study, we isolated for the first time MSC from tumor tissues of patients with head and neck squamous cell carcinoma (HNSCC). The isolated cells met the consensus criteria for MSC [35] and were successfully maintained and propagated *in vitro*. These tumor-derived mesenchymal stromal cells (TuMSC) exhibited a proinflammatory phenotype and responded to stimulation by tumor cells with a strong interleukin (IL)-8 release and with CD54 upregulation, respectively. Most importantly, TuMSC significantly enhanced the growth of human HNSCC lines when xenografted into recipient animals *in vivo*. Our data thus suggest a bidirectional interaction of MSC and tumor cells that, ultimately, results in the progression of HNSCC.

## Materials and methods

### Ethic statement

Human HNSCC tissue was obtained from surgical specimens after patients’ written informed consent using guidelines approved by the Ethics Committee of the University Hospital of Essen.

### Isolation of mesenchymal stromal cells

Head and neck cancer tissue samples were collected aseptically in sodium chloride (0.9%; Fresenius Kabi, Bad Homburg, Germany). Subsequently, tumor specimens were washed several times with Ringer’s solution (Braun, Melsungen, Germany) to remove the majority of erythrocytes. Tissues were cut into 1 to 2 mm pieces, washed extensively and digested in Ringer’s solution containing 5 mg/ml collagenase type II (CellSystems, Troisdorf, Germany) for 40 minutes at 37°C with gentle shaking. Tissues were centrifuged at 300 × *g* for 7 minutes, and the supernatant was discarded. The partially digested tissues were further treated with Ringer’s solution and 33 mg/ml dispase (Roche Applied Science, Mannheim, Germany) for 60 minutes at 37°C. The cell suspension was then centrifuged, and the pellet was resuspended in standard culture medium (high-glucose Dulbecco’s modified Eagle’s medium; Invitrogen, Karlsruhe, Germany) supplemented with 10% fetal bovine serum (Biochrom, Berlin, Germany), 1% penicillin/streptomycin (Invitrogen) and 1% sodium pyruvate (Invitrogen) and was transferred to tissue culture flasks. Nonadherent cells were removed by washing with phosphate-buffered saline 48 hours later and fresh medium was added to the remaining cells. During the culture period, cells were maintained at 37°C in a humidified atmosphere of 5% carbon dioxide. Cells were continuously passaged after reaching subconfluency by StemPro^®^ Accutase^®^ Cell Dissociation Reagent (Invitrogen) treatment for 5 minutes at 37°C. Bone-marrow-derived mesenchymal stromal cells (BMMSC) were obtained by a standard procedure as described previously [36].

### Flow cytometry

Direct immunofluorescence was performed for flow cytometric cell-surface marker immunophenotyping using the following specific monoclonal antibodies: CD14 PE (clone M5E2), CD19 PE (clone HD37), CD73 PE (clone AD2), CD90 (Thy-1) (clone 5E10) (all BD Bioscience, Heidelberg, Germany), CD34 fluorescein isothiocyanate (clone 581; Invitrogen/Molecular Probes), CD45 PE (clone 5B1; Miltenyi, Bergisch Gladbach, Germany) and CD105 fluorescein isothiocyanate (clone 166707; R&D Systems, Wiesbaden, Germany). To determine nonspecific signals, isotype controls were used at the same concentration as that used for the specific antibody. Analysis was performed using a FACS Canto II Flow Cytometer (BD Bioscience) and the resulting data were processed using Diva 6 software (BD Bioscience).

### Immunofluorescence microscopy

Cells were immunostained for Vimentin (1:200; mouse monoclonal clone VIM13.2; Sigma-Aldrich, Taufkirchen, Germany), S100A4 (1:100, rabbit polyclonal antibody; Abcam, Cambridge, UK), secondary antibody goat anti-mouse IgG fluorescein isothiocyanate (1:100; Dianova, Hamburg, Germany) and goat anti-rabbit IgG Cy3 bis-NHS ester (Cy3) (1:1,000; Dianova). Cells were examined with an Axioskop 2 microscope with a Ph2 Plan-Neofluar 20×/0.5 objective lens (Carl Zeiss MicroImaging, Göttingen, Germany). Images were generated using an Axiocam MRc microscope camera and Axiovision AxioVS40 Software (Carl Zeiss MicroImaging).

### Trilineage *in vitro* differentiation

Differentiation towards osteogenic, adipogenic and chondrogenic lineage was induced as described previously [37]. In brief, cells were seeded at a density of 3 × 10^3^ cells/cm^2^ on round glass slides in 12-well culture dishes (Greiner Bio-One, Frickenhausen, Germany). For osteogenic differentiation, cells were cultured for 21 days in Mesenchymal Stem Cell Osteogenic Differentiation Medium (PromoCell, Heidelberg, Germany). Medium change was performed every 3 to 4 days. Cells were finally stained with alizarin red S solution for 2 minutes to confirm the formation of calcium phosphate salts.

For adipogenic differentiation, we used Mesenchymal Stem Cell Adipogenic Differentiation Medium (PromoCell) for 14 days. To examine the generation of oil droplets in the cytoplasm after differentiation, cells were fixed with 10% formalin (Sigma-Aldrich) and stained with Sudan-III (Sigma-Aldrich) for 20 minutes at room temperature. Hematoxylin (Thermo Scientific, Bonn, Germany) was used to visualize nuclei.

Chondrogenic differentiation was induced after 48 hours of culture in standard medium supplemented with dexamethasone, 1 × 10^-3^ M l-proline (Sigma-Aldrich), 10 ng/ml transforming growth factor-β3 (Sigma-Aldrich) and 1% BD ITS Culture supplement (BD Bioscience). Medium change was performed every 3 to 4 days. To demonstrate the presence of glycosaminoglycans, Alcian blue staining was used. Dried 5 μm cryosections of the micromass pellets were fixed with formalin and washed with phosphate-buffered saline. Staining with Alcian blue 8GX (Roth, Karlsruhe, Germany) was performed at room temperature for 60 minutes.

### Cytokine profiling of mesenchymal stromal cells

After isolation and cultivation as described above, medium was exchanged and supernatant was collected from three different patient samples of MSC over a period of 24 hours. Cytokine secretion was analyzed with a bead-based multiplex assay (Bio-Plex; Bio Rad, Hercules, CA, USA) according to the manufacturer’s instructions.

### Stimulation of MSC with tumor-conditioned medium

TuMSC from different patient samples (see Table 1) were incubated in the presence of supernatants obtained from the HNSCC cell lines FaDu (ATCC HTB-43) and UM-SSC-22B (kindly provided by TK Hoffmann, University of Ulm, Germany). After 36 to 48 hours, tumor-conditioned medium was discarded and replaced by standard culture medium (high-glucose Dulbecco’s modified Eagle’s medium; Invitrogen) supplemented with 10% fetal bovine serum (Biochrom), 1% penicillin/streptomycin (Invitrogen) and 1% sodium pyruvate (Invitrogen). The control group was incubated for the same time period in standard culture medium. After 24 hours, medium was collected or cells were harvested. IL-8 secreted by activated MSC was measured by enzyme-linked immunosorbent assay (R&D Systems). Expression of CD54 (clone HA58; eBioscience, Frankfurt, Germany) on MSC was analyzed by flow cytometry.

**Table 1:** **Clinical information for tumor-derived mesenchymal stromal cell donors**

Patient number	Localization	T	N	M	Entity	Grading
1	Oral cavity	4	1	0	SCC	G3
2	Hypopharynx	4	0	0	SCC	G2
3	Oropharynx	2	1	0	SCC	G3
4	Oropharynx	4	3	0	SCC	G2
5	Hypopharynx	3	2	0	SCC	G2
6	Oropharynx	2	1	0	SCC	G2
7	Oropharynx	3	3	0	SCC	G3

G2, moderately differentiated cells; G3, poorly differentiated cells; SCC, squamous cell carcinoma.

### Murine xenograft model

Equal numbers of MSC isolated from three different patient samples were pooled and resuspended in fresh medium. TuMSC/BMMSC and HNSCC FaDu cells were mixed in a 1:1 ratio, resuspended in phosphate-buffered saline at a concentration of 10^6^ cells/100 μl and injected subcutaneously into the right flank of immunodeficient nude mice (TuMSC/BMMSC, *n* = 8/*n* = 4). A control group (*n* = 8) was injected with 0.5 × 10^6^ FaDu cells only. The tumor volume was calculated each day using the following formula:


where V is volume, w is width, and l is length. After 35 days (45 days for control group), mice were sacrificed and the tumors were analyzed. Early termination at day 35 for the MSC/HNSCC group was required by local animal ethics regulations. Animal experiments were approved by the responsible animal ethics committee of the state of North Rhine-Westphalia and carried out according to German guidelines for experimental animal welfare.

### Histology

Murine xenograft tumors were stained for Ki-67 and terminal deoxynucleotidyl transferase-dUTP nick end-labeling (TUNEL) as markers for proliferation rates and cell death. Anti-Ki-67 antibody MIB-1 mouse anti-human (1:100; Dako, Hamburg, Germany) was used as primary antibody, followed by peroxidase-conjugated rabbit anti-mouse (1:50; Dianova) and goat anti-rabbit (1:50; Dianova) secondary antibodies. Hematoxylin and eosin counterstaining was performed for nucleus identification. Frequency of Ki-67-positive cells as percent of total cells was calculated using three representative images per sample.

Staining for TUNEL was performed with the Apo-Direct Kit^©^ (BD Bioscience) according to the manufacturer’s instructions. The TUNEL-positive area was calculated with Image J (Fiji, open source, http://imagej.net/Fiji) software.

## Results

### Isolation and characterization of MSC from HNSCC tissues

**Figure 1:**
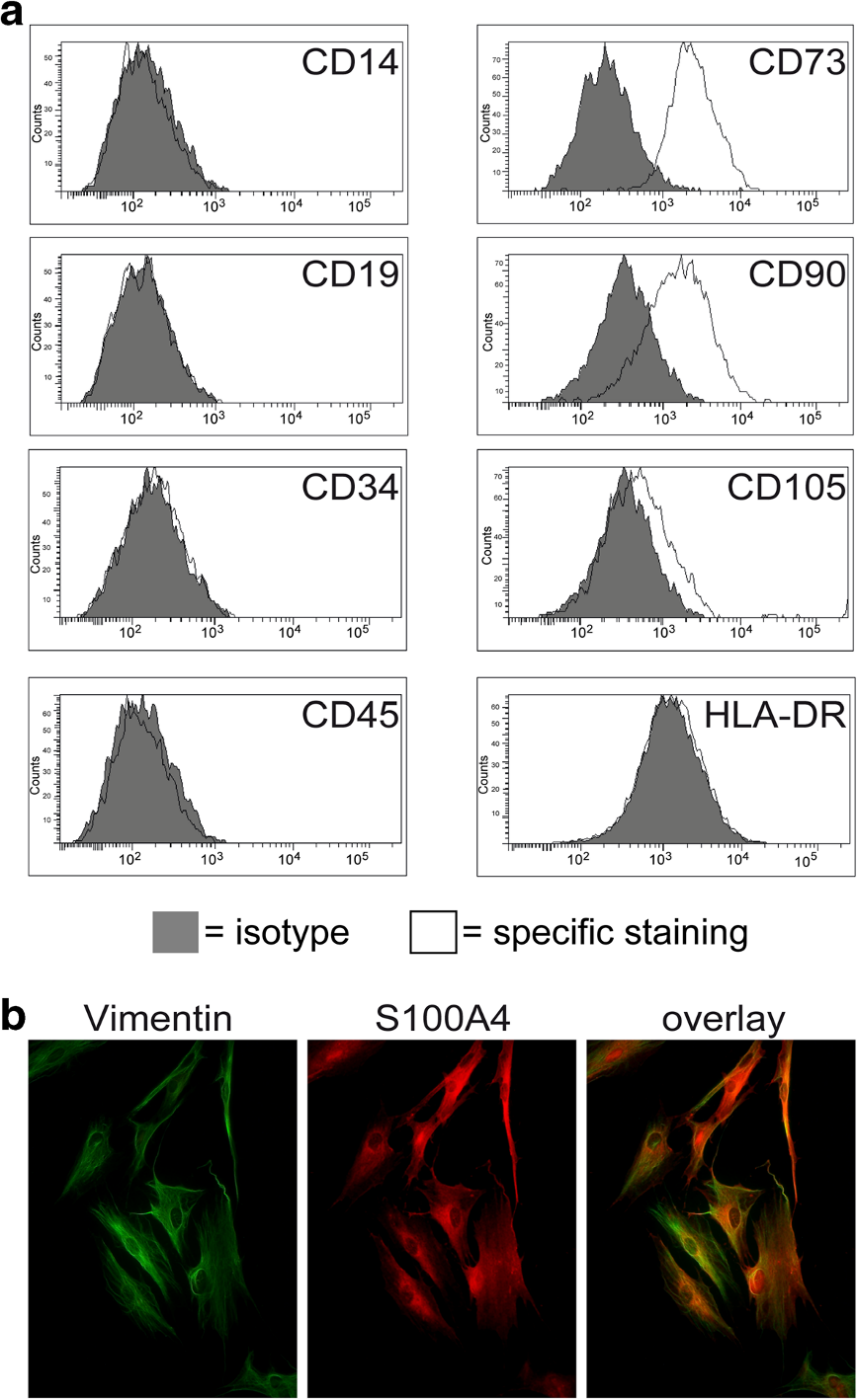
**Immunophenotyping of head and neck squamous cell carcinoma-derived mesenchymal stromal cells.** Mesenchymal stromal cells (MSC) were isolated from malignant tissue of patients with head and neck squamous cell carcinoma and were expanded. **(a)** Immunophenotyping was performed by flow cytometry. Data depicted as histograms. **(b)** Tumor-derived MSC were seeded on cover slips and immunofluorescence staining for vimentin and S100A4 was performed. Data and images from one representative experiment are shown.

**Figure 2:**
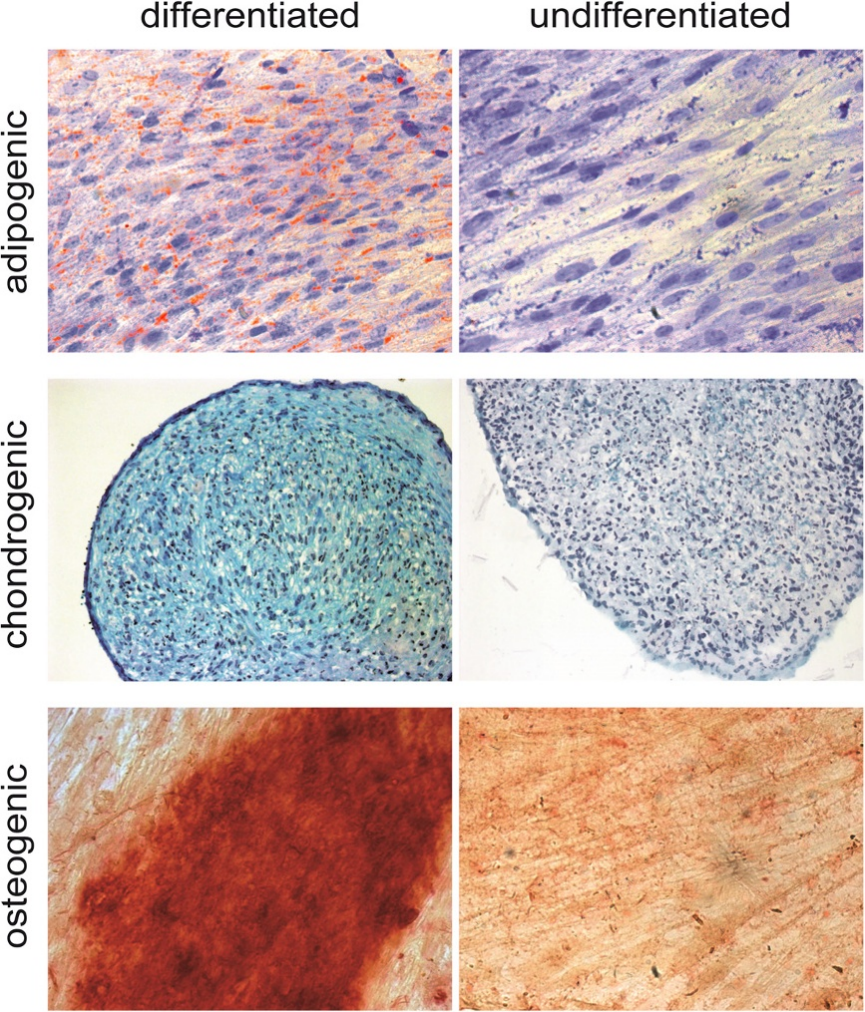
**Trilineage differentiation of head and neck squamous cell carcinoma-derived mesenchymal stromal cells.** Tumor-derived mesenchymal stromal cells were harvested and subjected to trilineage differentiation using standard differentiation-inducing culture conditions. Top row: adipogenic differentiation after 14 days, staining performed with Sudan III. Middle row: chondrogenic differentiation after 14 days, staining with Alcian blue. Bottom row: osteogenic differentiation after 21 days, staining with Alizarin red. Images from one representative donor are shown.

We first explored whether MSC can be obtained from malignant tissue of HNSCC patients employing standard isolation protocols for MSC. The isolated cells showed plastic adherence under standard culture conditions with a typical fibroblast-like morphology. To test whether the isolated cells expressed surface markers characteristic for MSC, these cells were subjected to flow cytometric analysis. We found that the isolated cells expressed MSC surface markers such as CD73, CD90 and CD105 and lacked the expression of hematopoietic markers such as CD14, CD19, CD34, CD45 and HLA class II (Figure 1a). Immunofluorescence analysis revealed the expression of further mesenchymal markers characteristic for MSC, such as vimentin and S100A4 (Figure 1b). An important criterion for MSC characterization is defined by the multilineage differentiation capacity. Under differentiation-inducing conditions, the isolated cells were tested for their chondrogenic, osteogenic and adipogenic multipotency. As shown in Figure 2, these cells could be induced to differentiate into all three lineages. Taken together, these studies established for the first time the expansion of MSC isolated from malignant tissue of patients with HNSCC.

### Cytokine profiling of TuMSC and modulation by tumor-derived factors

**Figure 3:**
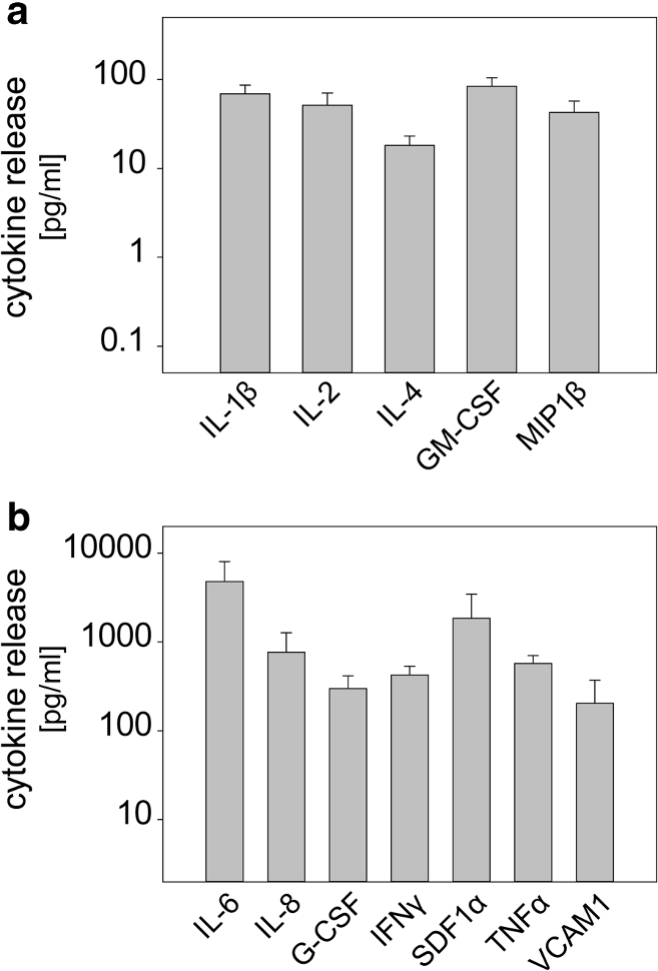
**Cytokine profile of tumor-derived mesenchymal stromal cells.** Tumor-derived mesenchymal stromal cells obtained from three individual patients were cultured for 24 hours and the culture supernatant was harvested. Concentration of cytokines was analyzed with a bead-based multiplex assay. Secretion was classified as no secretion (IL-5, IL-7, IL-10, IL-12, IL-13, IL-17; data not shown), **(a)** low secretion (<100 pg/ml) and **(b)** high secretion (>100 pg/ml). Data depicted as mean ± standard deviation. G-CSF, granulocyte colony-stimulating factor; GM-CSF, granulocyte–macrophage colony-stimulating factor; IFN, interferon; IL, interleukin; MIP-1 β, macrophage inflammatory protein-1β; SDF-1α, stromal cell-derived factor-1α; TNFα, tumor necrosis factor alpha; VCAM1, vascular cell adhesion molecule 1.

MSC exert their immunoregulatory activity partly via release of cytokines. Cytokine profiling of TuMSC revealed high constitutive expression of inflammatory cytokines (IL-6, IL-8, tumor necrosis factor alpha), homeostatic chemokines (stromal cell-derived factor-1α) and T-helper type 1 cytokines (interferon gamma) (Figure 3b). A number of other T-cell-modulating cytokines either were not expressed (IL-5, IL-7, IL-10, IL-12, IL-13, IL-17; data not shown) or were expressed at low levels (IL-1β, IL-2, IL-4, granulocyte–macrophage colony-stimulating factor, macrophage inflammatory protein-1β; Figure 3a).

**Figure 4:**
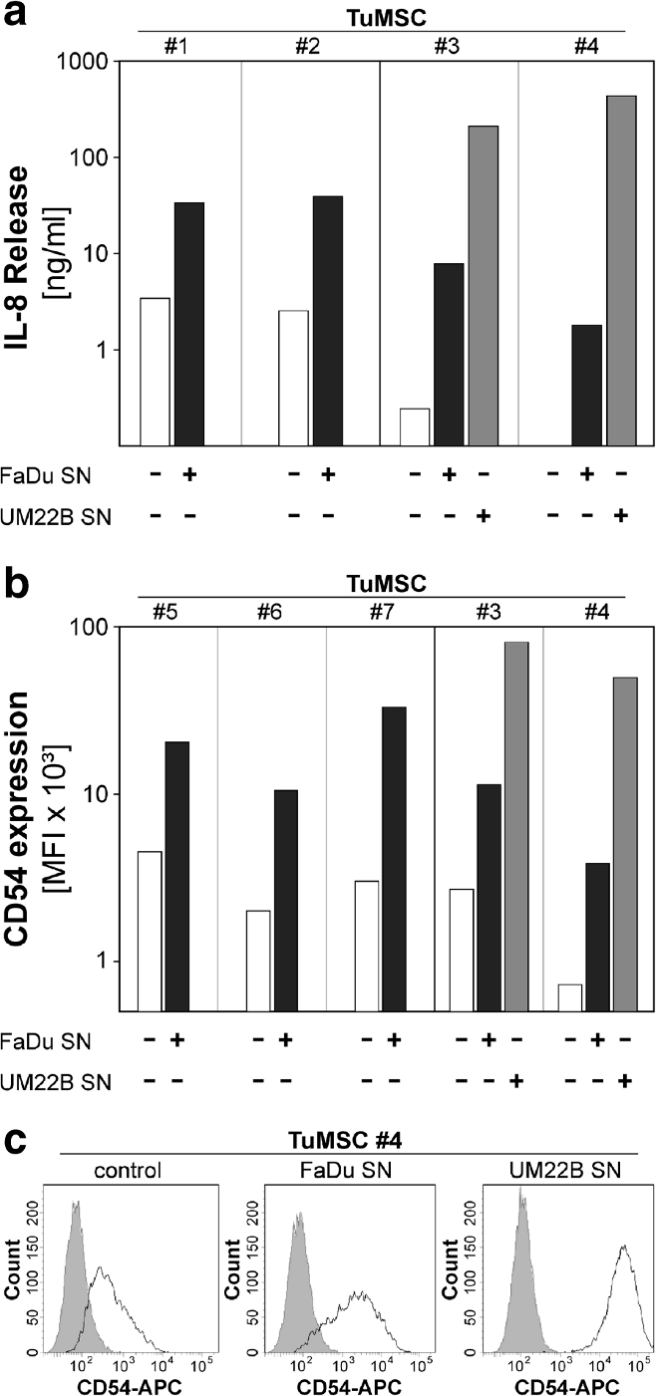
**Activation of tumor-derived mesenchymal stromal cells by head and neck squamous cell carcinoma-conditioned medium.** Individual tumor-derived mesenchymal stromal cell (TuMSC) lines were incubated in the presence of culture supernatants obtained from head and neck squamous cell carcinoma cell lines. After 36 to 48 hours, the FaDu/UM22B conditioned supernatant was aspirated and replaced by fresh standard culture medium. After another 24 hours, the TuMSC culture supernatant **(a)** or TuMSC **(b)** was collected. Concentration of interleukin (IL)-8 was measured by enzyme-linked immunosorbent assay **(a)** and expression of CD54 on activated TuMSC was analyzed by flow cytometry **(b, c)**. The experiment was performed with TuMSC from seven independent donors designated #1 to #7. APC, Allophycocyanin; MFI, median fluorescence intensity; SN, supernatant.

In the tumor microenvironment, TuMSC are likely to be engaged in cross-talk with tumor cells. To investigate a possible modulation of MSC by tumor cells, we exposed TuMSC to conditioned media obtained from the HNSCC cell lines FaDu and UM-SSC-22B and determined the subsequent release of cytokines. Specifically, we assessed the levels of IL-8 because this proinflammatory cytokine is known to be released by MSC from various sources and is involved in the modulation of the tumor microenvironment [38]. As illustrated in Figure 4a, HNSCC cell line supernatant strongly enhanced the release of IL-8 by MSC. The cell–cell adhesion molecule CD54 (ICAM-1) is a robust marker of activation in a large number of cell types. We therefore examined regulation of CD54 expression in TuMSC exposed to tumor-conditioned medium. Our results show that tumor-derived factors strongly upregulated surface expression of CD54 in all tested donors (Figure 4b) both for FaDu and UM-SSC-22B supernatant. As shown in Table 1, this activation occurred in MSC obtained from tumor samples of various histopathological background. In sum, these studies indicate that TuMSC from HNSCC patients release large amounts of immunomodulatory cytokines and are strongly activated if exposed to tumor-derived factors from HNSCC cell lines.

### Tumor-derived MSC promote HNSCC growth in a murine xenograft model

**Figure 5:**
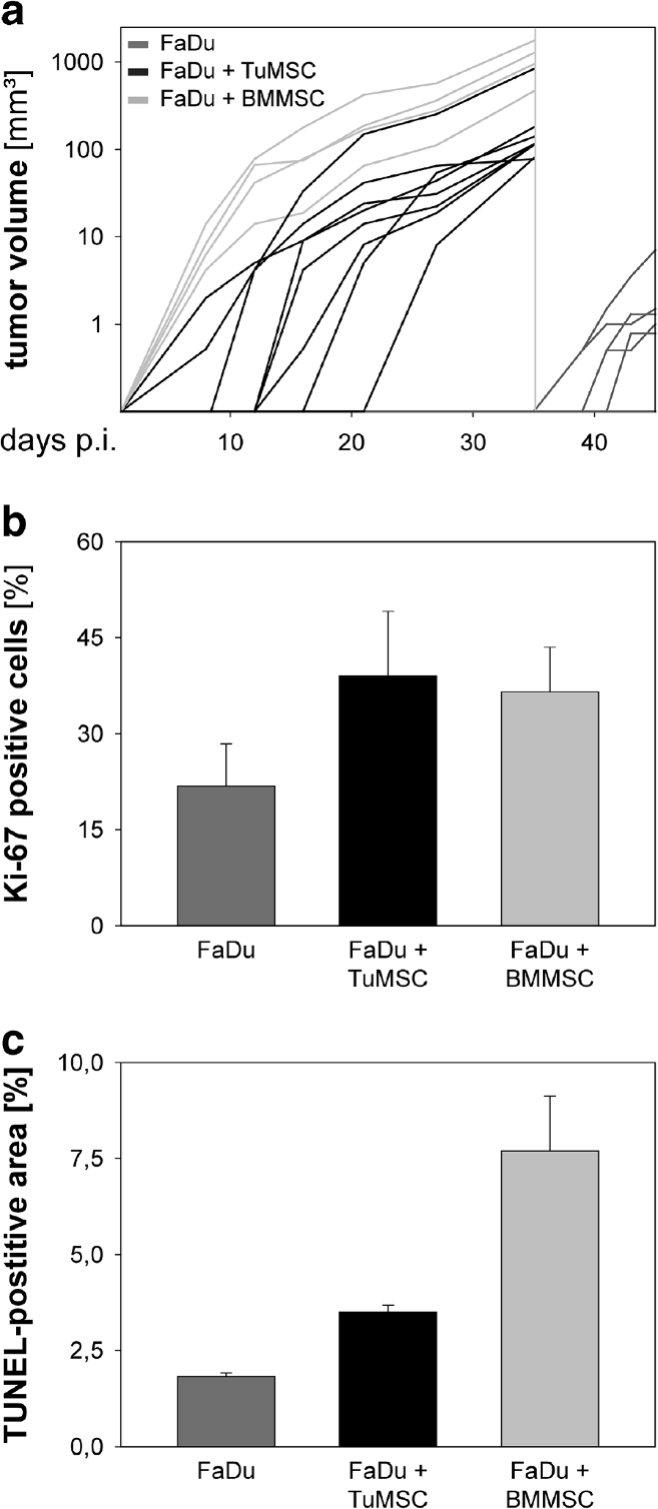
**Effect of mesenchymal stromal cells on head and neck squamous cell carcinoma progression in a xenograft murine model.** Equal numbers of mesenchymal stromal cells isolated from three different patient samples were pooled and admixed to HNSCC FaDu cells in a 1:1 ratio. Mixtures of tumor-derived mesenchymal stromal cells (TuMSC)–FaDu cells (*n* = 8) and bone marrow-derived mesenchymal stromal cells (BMMSC)–FaDu cells (*n* = 4) were injected into the right flank of immunodeficient nude mice. The control group was injected with only FaDu cells (*n* = 8). **(a)** The tumor volume was monitored by caliper measurements. Gray vertical line, sacrifice of mice according to ethic requirements. Cryosection slides of excised tumors (*n* = 3 for each group) were analyzed for Ki-67 staining **(b)** and apoptosis TUNEL staining **(c)**. Representative pictures were chosen and analyzed under a 400-fold magnification. p.i., post injection (of tumor); TUNEL, terminal deoxynucleotidyl transferase-dUTP nick end-labeling.

In the final part of the study we analyzed the impact of TuMSC on the progression of HNSCC *in vivo*. In this experiment we included BMMSC, for which tumor-promoting properties have been described in some models [39, 40], as a reference group. TuMSC/BMMSC were coinjected with HNSCC cells (FaDu) into immunodeficient nude mice. As a control, a group of animals was injected with HNSCC cells only. Tumor growth was assessed in a time course. The results show a significant difference in tumor growth between the three groups of mice (Figure 5). Specifically, the majority of mice injected with HNSCC cells and MSC developed palpable tumors already between days 7 and 20, and reached a high tumor volume around day 30. Owing to local animal ethics regulations, animals with FaDu/MSC tumors had to be sacrificed at day 35 because of tumor size and progression. In contrast, HNSCC cells lacking support by MSC displayed significantly delayed growth, with tumor growth starting around day 35 post injection. Histologic sections showed differences in proliferation rates and apoptosis between tumors coinjected with MSC and FaDu-only tumors. As shown in Figure 5b, coinjected tumors had a larger proliferative activity in comparison with FaDu-only tumors. This difference was also observed for TUNEL staining. These data demonstrate that primary MSC isolated from human HNSCC tissues and bone marrow provide substantial stromal support to promote HNSCC growth *in vivo*.

## Discussion

The histopathology of HNSCC is characterized by large stromal compartments, which surround the tumor islands. Only recently has it been fully recognized that the growth and progression of carcinomas are not only determined by the intrinsic properties of the tumor cells but critically depend on their interaction with the stromal cells. More recently, MSC have been recognized as important cellular components of the tumor stroma [18, 19]. As such, MSC can promote tumor development and progression by various means [12]. For instance, MSC directly modulate tumor cell biology and enhance proliferation, invasion and metastasis. MSC can also enhance angiogenesis by differentiating into or activating endothelial cells [13]. Finally, MSC have very potent immunoregulatory properties [41] and may downregulate antitumor immunity by a large variety of immunosuppressive mechanisms [14–16].

One way in which MSC interact with the surrounding cells in the tumor microenvironment is via release of cytokines and chemokines. Consistent with this idea, MCS isolated from our HNSCC patients released large amounts of cytokines. It is also noteworthy that, on a per-cell basis, this release may well exceed production of such cytokines by tumor-infiltrating leukocytes. Cytokine profiling revealed that IL-6, IL-8 and stromal cell-derived factor-1 were the most abundant cytokines secreted by the TuMSC in our study. Interestingly, this group of cytokines has previously been reported to be involved in the recruitment of MSC to the tumor site [8, 11, 42]. Still a matter of debate, however, is whether MSC are actively recruited to the tumor site or derive from tissue-resident precursors. While it appears to be unlikely that the entire stroma of carcinomas is actively recruited from a distant site, elegant studies by several groups indicate at least a partial contribution of bone-marrow derived cells to certain components of the tumor stroma [9, 20].

Irrespective of their origin, once present in the malignant tissue MSC are exposed to tumor cells and are likely to be engaged in a bidirectional interaction with those malignant cells. In support of this hypothesis, we found a dramatic upregulation of IL-8 release and of CD54 expression when TuMSC were exposed to tumor-conditioned medium. These findings are in agreement with a number of other studies demonstrating that MSC can be functionally modulated by tumor-derived factors [43–45].

During recent years, MSC have been isolated from a variety of tissues. Many studies analyzing the interactions of MSC with tumor cells used MSC derived from adipose tissue (a readily available tissue from which MSC can be easily isolated) or from bone marrow (where MSC were originally identified and characterized). While MSC derived from different sources share many common features and properties, some functional heterogeneity does exist depending on the tissue of origin [2, 3, 34]. This heterogeneity might also explain the contradictory findings regarding the effect of MSC on cancer progression [34]. Here, we investigated the effect of MSC derived from bone marrow and from malignant tissues of HNSCC patients on the biology of the tumor cells. We clearly showed that both types of MSC strongly promoted tumor growth when coinjected with HNSCC cells into recipient mice. Histologic observations showed histomorphological differences between the tumors coinjected with MSC and the FaDu-only tumors. Notably, coinjection of MSC results in a higher proliferation and apoptosis throughout the sections, indicating a stimulating and metabolism promoting role of MSC in the tumor microenvironment. Because very few studies have thus far analyzed human tumor-resident MSC, our findings might shed new light on how MSC modulate tumor progression in cancer patients.

## Conclusion

In this study, we isolated, expanded and characterized for the first time MSC from human malignant HNSCC tissues. This enabled us to assess basic biological functions of these cells, to investigate their modulation by tumor cells and to explore their effects on the growth of human cancer xenografts in mice. Our findings thus contribute to a better understanding of the tumor–stroma interactions and, ultimately, may stimulate the search for novel therapeutic strategies for HNSCC and other types of cancer.

## Abbreviations

BMMSC: bone marrow-derived mesenchymal stromal cells
HNSCC: head and neck squamous cell carcinoma
IL: interleukin
MSC: mesenchymal stromal cells
TuMSC: tumor-derived mesenchymal stromal cells
TUNEL: terminal deoxynucleotidyl transferase-dUTP nick end-labeling.
